# Dynamic Knee Valgus in Single-Leg Movement Tasks. Potentially Modifiable Factors and Exercise Training Options. A Literature Review

**DOI:** 10.3390/ijerph17218208

**Published:** 2020-11-06

**Authors:** Bartosz Wilczyński, Katarzyna Zorena, Daniel Ślęzak

**Affiliations:** 1 Centre of Rehabilitation and Training “Fizjo-world”, 81-537 Gdynia, Poland; 2 Department of Immunobiology and Environment Microbiology, Medical University of Gdańsk, Dębinki 7, 80-211 Gdańsk, Poland; kzorena@gumed.edu.pl; 3 Departament of Medical Rescue, Medical University of Gdańsk, Dębinki 7, 80-211 Gdańsk, Poland; daniel.slezak@gumed.edu.pl

**Keywords:** knee kinetics, knee abduction, single-leg squat, injury prevention, anterior cruciate ligament, sport performance

## Abstract

Dynamic knee valgus (DKV) as an incorrect movement pattern is recognized as a risk factor for lower limb injuries. Therefore, it is important to find the reasons behind this movement to select effective preventive procedures. There is a limited number of publications focusing on specific tasks, separating the double-leg from the single-leg tasks. Test patterns commonly used for DKV assessment, such as single-leg squat (SLS) or single leg landings (SLL), may show different results. The current review presents the modifiable factors of knee valgus in squat and landing single-leg tests in healthy people, as well as exercise training options. The authors used the available literature from PubMed, Scopus, PEDro and clinicaltrials.gov databases, and reviewed physiotherapy journals and books. For the purpose of the review, studies were searched for using 2D or 3D motion analysis methods only in the SLL and SLS tasks among healthy active people. Strengthening and activating gluteal muscles, improving trunk lateral flexion strength, increasing ROM dorsiflexion ankle and midfoot mobility should be taken into account when planning training programs aimed at reducing DKV occurring in SLS. In addition, knee valgus during SLL may occur due to decreased hip abductors, extensors, external rotators strength and higher midfoot mobility. Evidence from several studies supports the addition of biofeedback training exercises to reduce the angles of DKV.

## 1. Introduction

A key aspect of protecting people practicing both amateur and professional sport is injury prevention. For decades, researchers have been trying to identify modifiable risk factors and to create and implement interventions to minimize or eliminate these factors. An example of the work put into the research into the implementation of injury prevention is the model of Van Mechelen et al. 1992. Proposes four steps, the first concerns the determination of the order of magnitude of the problem in the population. The second step concerns the identification of the etiology and mechanisms of the injury. The next step is to introduce preventive measures to evaluate the effectiveness of the preventive measure in the final step [1,2]. Attempts to identify risk factors are due to the increase in the number of injuries such as the anterior cruciate ligament (ACL) rupture, and prevalent conditions such as patellofemoral pain (PFP) among athletes in recent years [3,4,5].

The complexity of the injury problem requires a multifactorial and multidirectional approach, bearing in mind injury exposure, which is often changing over time [6,7,8]. The coexistence of external risk factors such as training errors or the environment, and internal risk factors such as reduced muscle strength, and limited range of motion are also a challenge in this problem. To better control these factors, one must know the potential causes creating or predisposing their occurrence. It should be mentioned that one risk factor does not give complete certainty to the occurrence of an injury. However, interactions with other determinants allow the shaping of the profile of a person with the likelihood of injury [7].

### Dynamic Knee Valgus

**Figure 1:**
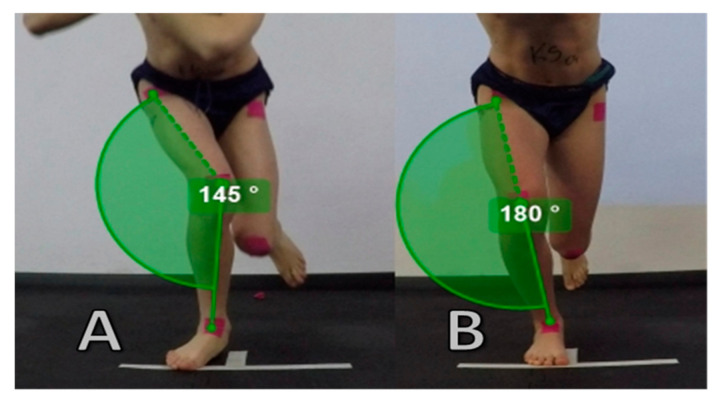
Example of dynamic knee valgus—knee move inwards from foot position (**A**) and correct movement pattern—knee under foot (**B**) during Single Leg Squat. Valgus quantitative assessment—angle between anterior superior iliac spine (ASIS), patella and center of malleolus (**A**)—145˚, (**B**)—180˚.

Dynamic knee valgus (DKV) is a movement pattern of the lower limb, potentially comprised of a combination of adduction and internal rotation of the femur, abduction of the knee, anterior tibial translation, external tibial rotation and ankle eversion [9]. During this pattern, medial knee displacement is observed, beyond the foot-thigh line, which indicates knee valgus movement [10]. DKV is one of the predisposing factors for acute and chronic injuries, such as non-contact ACL injury and the occurrence of PFP [11,12]. Both video analysis of athletes during injury games [13,14] as well as cadaveric studies [15,16] emphasize the mechanism of knee valgus as a mechanism of ACL injury. The differences in the above cases occur in the rotation of the tibia. Cadaveric studies show the mechanism of ACL strain during knee valgus with internal rotation [15,16]. The iliotibial band as an extension of the tensor fasciae latae and gluteus maximus muscles help to stabilize the knee joint in the frontal plane. However increased tension in tensor fascia latae can affect tibial abduction, resulting in knee valgus [17]. Moreover, in the Cadaveric study under non-weight-bearing conditions, it was proved that the greater the load on the iliotibial band, the greater the anterior tibial translation and the tibial valgus rotation increased, which may have an impact on dynamic knee valgus [18]. In addition, mechanoreceptors located in the tendons (Golgi tendon organ) and in the muscles (muscle spindles), which are responsible for the reflex function, assist in the positioning of the limbs [19]. Disturbances of these mechanisms, which are proprioceptive feedback system may contribute to ACL injuries [20]. Previous ACL trauma is a risk factor for recurrence in the same or the contralateral limb ranging from 6 to 32% [21]. Studies are suggesting that after the first ACL injury, an increased risk is associated with a change in movement control and an increase in joint load asymmetry, which may be one of the causes of the second ipsilateral or contralateral ACL injury [22]. Furthermore, dynamic valgus is responsible for patella maltracking, and in case of overload resulting from increased activity for example, it also increases stress on the patellofemoral joint and retinaculum causing PFP [23]. The example of the correct movement pattern and dynamic knee valgus is presented in Figure 1.

The authors, in their works on the knee valgus motion tasks, describe this inappropriate motor pattern as well as a knee medial foot position (KMFP), knee abduction or valgus knee excursion, differentiating in some cases peak or maximum valgus knee, and in landing tasks also detailing the assessment in the initial contact (IC) phase or the phase of maximum knee flexion [24]. The gold standard for the kinematic evaluation of the DKV pattern is the three-dimensional (3-D) method of analysis. Due to the lack of specialized equipment, a substitute, acceptable assessment method can be two-dimensional (2-D) analysis in the frontal plane motion [25,26]. Publications using these methods have been evaluated in the current review. The online databases from PubMed, Scopus, PEDro and clinicaltrials.gov, and physiotherapy journals and books were searched using keywords such as “dynamic knee valgus”, “knee abduction”, “knee kinetics”, “frontal plane projection angle” and “knee biomechanics” in combination with “injury prevention”, “exercise”, “risk factors”, “anterior cruciate ligament”, “patellofemoral pain”, “single leg squat” and “single leg landing”. Studies were included in the review only when dynamic valgus was assessed by the 2D or 3D method in SLS or SLL tests.

Despite evidence of DKV among patients with PFP [23,27,28] and after ACL injury [29], these studies were not considered in the review to focus on predictive values of healthy people. It is important to know the causes for the occurrence of DKV to help design an effective intervention to reduce the medial displacement of the knee.

Therefore, the purpose of the work is to conduct a review of the new literature regarding the potentially modifiable factors responsible for the dynamic knee valgus pattern in single-leg tasks. Furthermore, exercise intervention options for DKV reduction have been described.

## 2. Relationship between Squat and Landing on a Single-Leg and Double-Leg in Terms of Knee Valgus

The most common test for DKV assessment is the drop vertical jump test (DVJ), which involves a double-leg landing (DLL) [30,31]. Landing and squat testing tasks on both feet have gained interest over recent years due to research into the causes of ACL injuries [9,32]. The single-leg landing (SLL) and squats (SLS) were used to assess the biomechanics of the lower limb associated with ACL loading and the risk of PFP injuries [33,34]. Both squat and landing tasks consist of descending and ascending phases. They use the same movement pattern (i.e., flexion and extension of the torso, hips, knees and ankles in the sagittal plane). On the other hand, the squat, compared to landing, is characterized by a lower external load and slower movement, which seems to be an easier motor task to perform. It is worth paying attention to this because differences between these movement patterns can cause significant differences in the results for the knee valgus angles [29,35].

Considering knee valgus in these movement tasks, results may vary. There is evidence from research that confirmed the positive correlation of moderate to strong knee valgus between SLL, SLS and DLL [31,36,37]. However, in the study of Munro et al. 2017, the valgus angle of the FPPA was statistically significantly larger in the SLS test than in SLL (*p* < 0.001), and in SLS than in DVJ (*p* < 0.001) in the studied group of young basketball players [31]. Quite the opposite, Donohue et al. 2015, showed knee valgus results in 17 male and 17 female recreational athletes, where double-leg squat had the highest maximum valgus angles, followed by DLL, SLL, and the smallest SLS (*p* < 0.00001) [35]. Additionally, in the study of Taylor et al., 2017, comparing lower limb biomechanics in the DLL vs. SLL test in 15 recreationally active females, there were no differences in knee valgus angles (*p* > 0.05). Despite statistically significant changes in kinetics, differences were not visible for hip flexion, hip adduction, hip internal rotation, knee flexion or knee external rotation [37]. Due to the above conclusions from the research on knee valgus in landing and squat movement tasks, the authors of this review believe that single-leg tests should be separated from double-leg tests to better understand the causes of DKV.

The current review focuses solely on publications on single-leg movement tasks, mainly single-leg squat and single-leg landing in the sagittal plane for several reasons. First, knee valgus in the standing position on one leg with loss of balance is the main observed pattern during lower limb injuries [14]. Second, the vast majority of ACL injury cases occur during single-leg landing tasks [38,39,40]. Besides, single-leg tasks are more demanding movements than double-leg, which makes it easier to identify people with a higher risk of injury [10]. Finally, DKV is considered for single-legged tasks for specification and better practical implication.

## 3. Selected Modifiable Factors of Dynamic Knee Valgus

### 3.1. Impact of the Neuromuscular Trunk Control on Knee Kinetics

The “trunk dominance” term proposed by Hewett et al., 2009, as one of the causes of ACL injuries, is associated with dynamic knee valgus [41]. A lack of adequate neuromuscular trunk control and excessive lateral flexion of the trunk in the frontal plane predisposes to a shift in the center of gravity of the body away from the knee during squatting or landing. Shifting the center of gravity laterally to the knee joint is also associated with shifting ground reaction forces (GRF) in the same direction, which consequently causes knee valgus movement [42]. It was confirmed by Nakagawa et al. 2015, who observed an excessive ipsilateral lateral torso lean that was significantly correlated (r = 0.49, *p* < 0.01) with greater knee valgus in a group of thirty healthy participants. Electromagnetic sensors located on the sternum, sacrum and in the center of the hip allow the trunk angle to be determined during the SLS test [43]. For the above reasons, the hypothesis relating to the torso is taken into account when considering the causes of knee valgus in single-leg tasks. Neuromuscular trunk control is inseparably linked to the muscles of the torso. Core muscles refer, among others, to the stabilizing forces generated by the muscles of the hip, pelvis, and torso. When the body is loaded on one leg, hip abductor muscles work with the quadratus lumborum muscle to keep the position of the pelvis horizontally and prevent excessive adduction of the femur [44]. There is evidence that in the case of greater trunk lateral flexion muscle strength in side-lying planks, participants of both sexes show lower dynamic knee valgus angles in SLS [43,45,46]. The characteristics of studies in causes of dynamic knee valgus are presented Table 1. However, the large complicity of muscles of the hip abductors supporting the isometric lateral trunk flexion should be emphasized [47]. There is a need for more research on the effect of torso muscle strength on DKV. However, current publications suggest that the weak lateral trunk flexor muscles appear to be one of many predisposing factors for DKV in SLS.

### 3.2. Hip Strength and Muscle Activation as Causes of the Incorrect Movement Pattern of the Knee

The hip works as a proximal mechanism playing a key role in the neuromuscular control and compensation of excessive movements of the lower limb kinematic system [48]. Movement of the hip joints depends mainly on the work of the gluteus muscles. When considering the functions of the gluteus, it should be noted that they work in contradiction to the dynamic knee valgus movement by hip abduction and external rotation [49]. In addition, weakened hip abductors may affect the compensatory movement of pelvic drop and lateral torso displacement [50]. To date, several studies have shown a link between the occurrence of dynamic valgus in single-leg tasks and weak hip strength, mainly among female participants for abductors [46,51,52,53,54,55], external rotators [45,46,52,53] and extensors [46,52], but also in males for abductors [51,52,53] external rotators [45,52,53] and extensors [52,53]. However, some studies are of the opposite position, confirming the belief that evidence of the relationship between hip strength and DKV is conflicting [56,57]. The relationship between hip muscle strength and dynamic knee valgus undoubtedly depends on movement tasks that are used to assess valgus, i.e., ballistics, running, walking, cutting, squats, single-leg, double-legged. Different methods of assessing muscle strength also affect different results. Among other reasons, a systematic review of Cashman et al., examining the effect of weak hip muscles on DKV was unable to determine definitive conclusions or clinical recommendations [58]. Cronstrom et al., showed no relationship between hip strength (hip abduction, extension, external rotation) in women during various functional motor tasks [47]. A significant association (r = −0.38, 95% CI - 0.53 to –0.21, *n* = 114) was revealed between the lower hip external rotator strength and greater peak knee valgus angle in a subgroup of SLS test.

This problem was noticed by the team of researchers Dix et al., in their systematic reviews and meta-analysis, specifying the subgroups and revealing the link between hip muscle strength and knee valgus in ballistic SLL tasks, and to minor size SLS and not in ballistic double-leg tasks. The decreased strength of hip abductors, extensors and external rotators was associated with DKV during single-leg drop landings among subgroups of healthy women (*p* < 0.05). The difference in the results of the kinematic evaluation of the lower limbs between the test tasks of single-leg and double-legs is also due to different demands and muscle recruitments, which, among others, require greater eccentric work to provide control in the frontal plane angles [57]. During single-leg motor tasks, more eccentric work is required of the standing leg hip abductors to resist the contralateral pelvic depression and femoral adduction.

This thesis is confirmed by the latest study, which showed a strong negative correlation between knee abduction angle and concentric strength (r = −0.55. *p* = 0.02, R^2^ = 0.3) and also eccentric (r = −0.56. *p* = 0.01, R^2^ = 0.31) hip abductors in SLL tests among active women. In SLS in the same group there was a negative correlation between the knee valgus angle and hip abduction concentric strength (r = −0.55, *p* = 0.02, R^2^ = 0.3) and hip extension eccentric strength (r = −0.5, *p* = 0.04, R^2^ = 0.23) [53].

#### Activation and Coactivation of the Gluteus Muscles for Knee Control

Considering in more detail the muscle activation and strength, two muscles should be specified: Gluteus Medius (abductor) and Gluteus Maximus (extensor and external rotator). The approach by Naematallah et al., to the problem of the activation and strength of the hip concerning knee valgus is a relevant exploration of the topic. EMG activation was performed for men and women in various single-leg movement tasks (SLS and SLL in three directions). In the female group during SLL (Forward Landings) there was a correlation (r = 0.56, *p* = 0.01, R^2^ = 0.31) between gluteus maximus EMG activity and knee abduction angle. In the male group during SLS, gluteus medius EMG activity was strongly correlated (r = 0.65, *p* = 0.004) with the knee abduction angle [53]. However, the results show a positive correlation that suggests increased EMG activity along with increased valgus angles. This may indicate a heightened neural drive mechanism to increase muscle fiber recruitment in motion control for participants with lower hip strength [53,59].

Coactivation of muscles is an important mechanism in the stabilization and motor control of the lower limb. The condition of this phenomenon is the simultaneous contraction of agonists and antagonists of a given joint [60]. Evidence from Mauntel et al., allows us to bend the effect of gluteal muscle coactivation with hip adductors. Muscle coactivation ratios calculated by multiplying the average muscle activation of gluteus medius and maximus and the average activation of hip adductor (designated as GMax: Hip Add and GMed: HipAdd) were used in the study. During the descent phase of SLS the mean EMG amplitude was normalized to maximal voluntary isometric contraction (MVIC). Participants in the valgus group showed significantly (*p* < 0.05) smaller GMax: HipADD (2.4 ± 1.1), and GMed: HipADD (1.1 ± 0.62) ratios than the control group (4.5 ± 3.9 and 2.4 ± 1.8) Thus, EMG hip adductor activation was greater than EMG activation of the gluteus maximus and minimus in the valgus group compared to the control group [10].

In summary, there is some evidence to support the thesis about the influence of the strength of hip abductors, extensors and rotators in SLL tasks in women. In the SLS test, the relationship is conflicting. Coactivation of the gluteus muscles with hip adductors also seems to be important. For men, the evidence is less numerous and requires future research. However, it is worth considering adding to the prevention of lower limb injuries, and to training to improve the DKV pattern, and exercises strengthening the gluteal muscles for both women and men.

### 3.3. The Role of Strength and Muscle Activation Quadriceps and Hamstring in Stabilizing the Knee

The role of quadriceps and hamstrings in stabilizing the knee seems to be extremely important from an anatomical point of view. Both muscles are knee stabilizers, pressing the joint surfaces during movement. The hamstring and quadriceps muscles, thanks to their distal tibial attachments, counteract excessive rotational movements and abduction of the knee [61,62]. Several studies have revealed the relationship between knee flexor and knee extensor in valgus knee positioning in the SLS test [45,51,61] Claiborne et al., found a weak to moderate negative correlation knee flexion (r = –0.43, *p* < 0.001) and knee extension (r = –0.37, *p* < 0.05) showing that participants with greater knee strength exhibited less valgus motion. There is also evidence of the impact of concentric hamstring strength from a study in girls aged 10–13 (pubertal onset). The reduced concentric hamstring strength affected (*p* < 0.05) the reduced ability to control the lower limb, which increased knee valgus values during a jump and SLL [61]. In contrast, a weak positive correlation (r = 0.33, *p* = 0.02) marked in the research of Wilson et al., showed that the greater strength of the knee flexors, the greater the knee valgus angles [45]. A meta-analysis of the three above-mentioned studies (96 healthy participants in total) by Cronstrom et al., showed no evidence for the association of hamstring strength with a greater peak valgus moment (r = −0.20; 95% CI −0.54 to 0.19) [39]. There are hypotheses regarding support for knee joint stabilization in Varus-Valgus moments by quadriceps-hamstring muscle co-contraction. Co-contraction is used to stiffen a joint to control the position of the limb [62,63]. Probably, the mechanoreceptors lying in the periarticular structures, including the collateral ligaments of the knee, are responsible for the reflex tension of the lateral and medial muscles of the knee, thus counteracting valgus movements [63].

Increased pre-activity of the vastus lateralis muscle and decreased pre-activity of the vastus medialis predicts increased knee valgus in the jump and SLL in a female. Twenty-eight volunteers participated in a 3-D motion capture kinematic study assessing knee valgus during a jump and landing on one leg. In addition, participants had bipolar surface EMG electrodes synchronized with a motion capture system in order to assess the activity of the lower limb muscles (rectus femoris, vastus lateralis, vastus medialis, medial hamstring and lateral hamstring). The partial regression coefficients used (b) revealed an association between muscle activity and valgus in women. A greater knee valgus angle was associated with greater muscle pre-activity of the vastus lateralis (b = 1.397, *p* = 0.013) and lateral hamstring (b = 1.760. *p* = 0.008). A smaller valgus angle was associated with increased pre-activity of the vastus medialis muscle (b = 2.197, *p* = 0.009). There was no association between variables for both sexes together and for men separately (*p* ≥ 0.05) [64].

These studies support the hypothesis that muscular imbalance in medial-to-lateral quadriceps-hamstring co-contraction may contribute to increased knee valgus among recreationally active women [64]. Contrary to the above results, there is a study assessing knee valgus during SLL in thirty-five female athletes. EMG assessment of muscle pre-activity of the vastus lateralis (VL), rectus femoris, lateral hamstring (LH) and VL: LH ratio was not a significant predictive (*p* > 0.05) factor for knee abduction [65]. Despite the hypotheses and recent studies regarding knee muscles and DKV, the evidence is conflicting.

### 3.4. The Possible Impact of the Ankle Range of Motion and Foot Aspect

Considering the lower segment in the kinematic system of the lower limbs, it is impossible not to mention the role of the ankle and foot in the context of DKV.

Movement tasks consisting of squat or landing require, inter alia, flexion movement in the sagittal plane in the hip and knee joints and dorsiflexion in the ankle joint. If there is a range of motion (ROM) deficit in one segment of the lower limb system, there is usually compensation in the transverse and frontal planes. This situation can occur when the ROM of the ankle dorsal flexion is limited, which can force compensated movement of excessive pronation of the ankle, internal tibial rotation, adduction and internal rotation of the thigh and pelvic drop, which in turn leads to DKV [66,67,68]. Seven studies included in the meta-analysis of the researchers, Lima et al., allowed the publication of the claims, saying that the DF ROM restriction is associated with DKV (SMD −0.65, 95% CI −0.88 to –0.41), regardless of whether DF ROM was measured in a situation of foot loading (weight-bearing) or not (non-weight bearing), also with knee flexion or extension [68]. However, three studies in the meta-analysis were based on the lateral and forward step-down tasks [61,62,63], and only one study focused on the SLS test [12]. Evidence supporting the impact of the DF ROM restriction in the SLS test showed studies by Wyndow et al., and Mauntel et al. In both cross-sectional studies, 70 healthy subjects of both sexes participated [12,67]. In the study of Mauntel et al., DF ROM was examined with a goniometer in the 90° knee flexion and knee extension in a supine body position. The valgus group showed 37.5% (extended) and 33.1% (flexed) smaller DF ROM than the control group [12].

In the second study, the authors examined DF ROM in a wall test. The maximum distance of the foot (longest toe) from the wall that allows the knee to touch the wall without lifting the heel. The mean of DF ROM was (10.7 cm ± 3.4 cm), where the mean peak FPPA valgus (4° ± 7°). DF ROM was associated with valgus (Odds Ratio (OR) 1.8, 95% CI 1.2 to 2.9). A greater indicator of OR for ankle joint dorsiflexion indicates that a lower range of ankle contributes to a greater FPPA [67]. Moreover, participants with greater midfoot mobility are 2.4 more likely to achieve a greater peak valgus knee angle during SLS. Where mobility was measured as the difference in midfoot height in an unladen weight and the difference in midfoot width in the same way [67]. Pronation occurring as compensation at the moment of limiting the knee forward movement relative to the foot during squatting tasks occurs especially in midfoot and the subtalar joint [69]. Rear-foot eversion and excessive pronation can cause tibial internal rotation. This may result in a change in the kinematics and kinetics of the lower extremities during motor tasks [54,70]. In one study, one hundred and thirty female basketball players were tested for knee valgus by the 2-D method. The valgus was assessed as Knee-in distance (KID). It was the distance measured in centimeters from the big toe to the point where the line connecting the center of the patella with the anterior superior iliac spine falls to the ground. The heel-floor test (HFT) assessing rear-foot kinetics was positive when the eversion rear-foot angle reached ≥5˚ in SLS and SLL tests. Participants with a positive HFT showed higher valgus values than participants with a negative HFT in SLS (12.2 ± 5.1 cm, vs. 8.7 ± 5.2 cm) and in SLL (14.7 ± 7.2 cm vs. 8.9 ± 5.5 cm) [54]. The characteristics of studies in causes of dynamic knee valgus is presented Table 1.

There is evidence from two studies confirming the effect of the DF ROM restriction on DKV in SLS. More research comes from studies evaluating kinematics in the double-leg squat and lateral or forward step-down tests.

### 3.5. Fatigue as a Factor in the Context of Dynamic Knee Valgus

The problem of fatigue as a factor influencing the change in knee biomechanics has been studied for years [38,71,72]. Simplifying the definition, fatigue can be described as a reduction in the underlying physiological and psychological function, causing a change in movement and manifesting itself in altering movement patterns [7]. Fatigue can be divided into two mechanisms, central and peripheral. Central fatigue refers to reduced muscular activity, while peripheral fatigue refers to the reduced ability to generate strength. Both are characterized by a decrease in neuromuscular control properties [73]. Various fatigue protocols were used by researchers. Some researchers used exercises such as running on the treadmill, squats or jumps to provoke the central mechanism of fatigue [72,74,75]. When triggering a peripheral mechanism, researchers used resistance exercises directed at the local joint, e.g., flexion-extension in the knee joint [72]. Studies on the effects of fatigue on knee valgus among athletes show conflicting results. Santamaria et al., in their review showed the effect of fatigue protocols on increasing knee angle in SLL only when it was an unanticipated task [38], a systematic review of Barber-Westin et al., showed no difference in research on the effect of fatigue on knee kinematics (knee abduction and internal rotation) in single-leg tasks, both in planned and unplanned tasks (*p* > 0.05) [76] and finally, results that are not statistically significant on the overall fatigue effect on knee valgus in SLL in the latest comprehensive review (*p* > 0.05) [6]. The explanation for such results may be a non-standardized fatigue protocol and the provision of too low a load protocol for athletes. 

The mechanisms of proprioception mentioned in the previous sections are responsible for the stabilization of the joints. Mechanoreceptors with distinction Golgi tendon organs and muscle spindles play an important role [19,77]. Muscle fatigue can reduce proprioceptive abilities anywhere along the pathway associated with muscle contraction [77]. Thus, fatigue by impairing the neuromuscular control of the lower limb may lead to impaired ability to dynamically stabilize the knee, including positioning the knee in the axis [77,78].

Neuromuscular fatigue is often mentioned as a modifiable risk factor for ACL injury. The hypotheses are related to the effect of fatigue on the altered pattern of muscle activation, reduction in muscle strength or increased ground reaction forces, [6,71,76,79]. In addition, fatigue pathways run parallel to injury pathways, which lead to a decreased performance by athletes, [71,80]. In contrast, Bourne et al. 2019, presented reviews showing no relationship between workload and the occurrence of ACL injury, highlighting deficiencies in quantifying fatigue parameters in prospective studies [81].

## 4. Exercise Training to Reduce Dynamic Knee Valgus

Exercises with the addition of real-time visual biofeedback can have a positive effect on the immediate reduction of DKV. Twenty-four women with observed MKD participated in the Marshall et al. 2020 study were assigned to the feedback group and control group. Kinematic evaluation of the lower limbs in the SLL test was performed using a 3-D analysis system. The intervention consisted of 1 session in which four exercises (SLS, double-leg squat, single-leg, and lateral step down) were performed by both groups in a mode of 10 repetitions each. Visual biofeedback in the feedback group consisted of performing exercises with an observation of its movement pattern on the monitor. The software responsible for the analysis of movement showed colors following the axis of the knee. The participant had to keep the color green, which appeared when the knee abduction angle was <5°. The feedback group decreased the knee abduction angle from preintervention (7.95° ± 3.80°) to postintervention (4.93° ± 1.64°) (*p* = 0.008) and decreased the angle by 6.16° more than in the control group in the landing phase IC [82].

The importance of the impact of the hip abductor muscle strength for DKV has been described in previous sections of this review. Palmer et al., attempted to evaluate five weeks of isolated hip abductor (side-lying hip abduction exercise with ankle weights) training in one group and functional motor exercises (from double-leg to single-leg squatting) in the second group in 29 healthy participants with observed knee valgus. A DKV reduction of 10° occurred in the group performing functional exercises and by 5° in the group strengthening the hip abductor in the SLS test. Despite clinically significant changes (clinically significant defined as a 3° reduction [77] in knee kinematics), the results were not statistically significant (*p* > 0.05) [83].

Also, in the Hopper study, despite the reduction of knee abduction in SLL (training group reduced DKV by 7.74° ± 7.42° compared to control group by 3.21° ± 5.66° at maximum knee flexion angle), the results were not statistically significant (*p* > 0.05). Of the twenty-three netball women, thirteen performed extensive neuromuscular training, and 10 were allocated in the control group without intervention. The six-week training consisted of several exercises divided into three stages (warm-up, plyometrics, strength) [84].

Statistically significant results for the reduction of valgus (*p* = 0.001) measured as FPPA in the SLS test were obtained by Dawson and Herrington’s study. For six weeks, three times a week, gluteus medius and maximus training (side-lying hip abduction, quadruped leg extension, clam exercise and front step-up) were performed for the strength group and biofeedback training (SLS exercise with observation and correction of the technique in front of the mirror) for skill group. In both groups, there was a significant improvement in FPPA and remained at a similar level after 12 weeks from the value before training. DKV was reduced in the strength group from (12.76° ± 4.44°) to (7.28° ± 5.10°) and skill group (13.34° ± 4.46°) to (8.81° ± 4.22°). The biggest change occurred in women in the skill group with feedback training with an average of 8.1°. However, only 17 recreationally active participants took part in the study (strength group (four men, five women) and skill group (four men and four women)), which is a very small sample [85].

In addition, core-muscle training lasting eight weeks compared to the control group without intervention significantly influenced the reduction of the angular value of knee valgus in the SLS test in female basketball players. The valgus decreased from 5° (IQR = 2.0°–6.1°) to 2.2° (IQR = 0.9°–3.2°) (*p* = 0.008). No changes were observed in the control group. Core-muscle training performed on average 4.7 times a week consisted of three exercises (Nordic Hamstring, Side Plank, Front Plank) with individual intensity progression. It is worth noting that the same program did not change knee valgus angles in the double-legged DVJ test. This study also had a small sample size (*n* = 17) [86].

The combination of many types of exercises with an emphasis on the quality of movement control and the alignment of the limbs and torso, thus avoiding knee valgus contributes to the reduction of the risk of injury in sports populations [87,88]. The research team of Attwood et al. 2018, by introducing an Injury Prevention Exercise Program, which consisted of exercises to improve balance, proprioception, landing and cutting skills, and strengthening exercises showed a reduction in injuries among rugby players. Exercise progression focused, among other things, on changes in time, intensity, change of direction of movement, and by removing a visual component. Plyometric exercises changed consistently from simple double-legged tasks to demanding single-legged tasks. Feedback was used for participants in the form of information about the quality of movement and body alignment. The proposed program lasted 42 weeks, three times a week. The set consisted of a warm-up (5–10 min) and the main part (15 min) and was performed by 41 male adult rugby union clubs. Compared to the control group, the results showed a 40% reduced incidence of lower limb injuries. Including injuries of the knee ligaments, where the injury incidence rate in the control group was 1.24 (0.77–2.01), and in the intervention group was 0.61 (0.31–1.19) (90% CI) [87].

Exercises focused on muscle strengthening, balance and plyometric training, with lower limb positioning feedback (e.g., avoiding valgus) may be beneficial in reducing the risk of lower limb injuries.

## 5. Conclusions

DKV is a leading movement pattern that can be responsible for knee injuries to recreational and professional athletes. The above review focused on modifiable factors, with a look at particular segments in the kinematic chain and the role of fatigue in single-leg tasks. Evidence suggest that decreased mainly hip abductors, but also extensors, external rotators strength and higher midfoot mobility are potential factors predisposing to knee valgus during SLL. Strengthening and activating gluteal muscles, improving trunk lateral flexion strength, increasing ROM dorsiflexion ankle and midfoot mobility should be taken into account when planning training programs aimed at reducing DKV occurring in SLS. Evidence from one study suggests excessive rear-foot eversion as a cause of knee valgus in both SLS and SLL tests among young basketball players. There is some evidence from studies showing exercise training consisting of gluteal strengthening, biofeedback training, and a core-stability workout can improve the dynamic knee valgus pattern in healthy adults. It is worth considering the above reports in the context of selecting exercises for injury prevention programs.

A lower ratio of gluteal muscle co-activation to hip adductors and reduced mobility of the ankle and foot have been observed in people with DKV. Considering this evidence, it can be concluded that by reducing the tension or stretching of the hip adductors and plantar flexors of the ankle, we can reduce knee valgus angles.

Exercise programs to strengthen the muscles (with an emphasis on gluteal muscles), improve balance, core-stability and plyometric, with biofeedback on lower limb position, thereby avoiding knee valgus, provided by physicians, physical therapists and trainers, can reduce the angle of knee valgus and thus reduce the risk of lower limb injuries. 

The vast majority of research into the causes of DKV is assessed in the female population. It should be noted that women show greater values of DKV than men. Although the double-leg tasks are the most common DKV assessment tests, lower limb injuries most often occur when landing on one leg. There is a significant difference in the shift of the center of gravity towards the opposite side from the SLL, which forces one side to engage more strongly. In the case of double-leg landing, the involvement is distributed bilaterally.

Therefore, research must focus more on the tasks of a single-leg rather than the double-legs. Further work is needed to determine causes for the occurrence of and exercise training for reducing DKV in single-leg tasks, especially in SLL.

## Figures and Tables

**Table 1:** Characteristics of studies in causes of dynamic knee valgus (DKV).

TRUNK			
Study	Participants	Outcome parameters	Results
Wilson et al. 2006	F = 22 (19.4 ± 0.7y) M = 24 (19.9 ± 2.3y) Division 1A or 1AA basketball, soccer, or volleyball players.	Peak isometric torque: trunk flexion, lateral flexion and extension Knee valgus: FPPA (2-D) during SLS	BOTH: SLS Greater trunk lateral flexion strength among participant with lower knee valgus angles (FPPA)
Stickler et al. 2015	F = 40 (22.88 ± 0.32y)	Handheld dynamometer isometric: side lying plank test (trunk lateral flexion) Knee valgus: FPPA (2-D) during SLS	FEMALE: SLS Greater trunk lateral flexion strength among female with lower knee valgus angles (FPPA)
Nakagawa et al. 2015	F = 20 M = 10 (both: 22.3 ± 3.0y) Control group (healthy without PFP)	Handheld dynamometer isometric: trunk extension, flexion with rotation and side bridge Knee valgus: Electromagnetic tracking system (3-D) during SLS	BOTH: SLS Greater strength of trunk lateral flexion (side bridge test) among healthy participant with lower knee valgus angles.
KNEE			
Study	Participants	Outcome parameters	Results
Wilson et al. 2006	F = 22 (19.4 ± 0.7y) M = 24 (19.9 ± 2.3y) Division 1A or 1AA basketball, soccer, or volleyball players.	Peak isometric torque: Knee flexion and extension Knee valgus: FPPA (2-D) during SLS	BOTH: SLS Greater strength of knee flexor among participant with higher knee valgus angles (positive correlation)
Claiborne et al. 2006	F = 15 (23.5 ± 3.7y) M = 15 (26.4 ± 5.2y)	Isokinetic eccentric/concentric strength: knee extension and flexion Knee valgus: FPKM (Frontal plane knee motion) in 3-D during SLS	Both: SLS Participants with greater knee strength exhibited less valgus motion
Wild et al. 2013	F = 33 (10–13y, Tanner stage II)	Isokinetic eccentric/concentric strength: knee extension and flexion Knee valgus: 3-D motion analyses system during SLL	Female: SLL Females with lower flexor (hamstring) strength displayed significantly greater knee valgus angles.
ACTIVATION			
Palmieri-Smith et al. 2008	F = 18 (24.0 ± 5.2y) M = 10 (23.6 ± 3.8y) recreationally active (Tegner score 5 or 6).	EMG: Dynamic and MVIC rectus femoris, vastus lateralis, vastus medialis, medial hamstring, lateral hamstring Knee valgus: 3-D motion capture kinematic during SLL	Female: SLL A greater muscle pre-activity of vastus lateralis and lateral hamstring was associated with greater peak knee valgus angle. A smaller peak valgus angle was associated with increased pre-activity of the vastus medialis muscle Both or males: SLL Muscle activation was not associated with the peak knee valgus angle
Brown et al. 2013	F = 35 (15.1 ± 1.2y) basketball, field hockey, and soccer players	EMG: Dynamic and MVIC vastus lateralis (VL), rectus femoris, lateral hamstring (LH) Knee valgus: 3-D motion analyses system during SLL	Female: SLL Muscle pre-activity of vastus lateralis, rectus femoris, lateral hamstring, and VL: LH ratio was not a significant predictive.
HIP			
Study	Participants	Outcome parameters	Results
Neamatallah 2020	F = 17 (25.7 ± 4.5y) M = 17 (26.9 ± 3.8y) physically active (participating in least 3 h of exercise per week)	Isokinetic muscle strength concentric/eccentric: hip abduction and hip extension Knee valgus: 3D motion analysis during SLS/SLL (Forward Landing - FL, Single Medial Landing -SML, Single Lateral Landing - SLL)	FEMALE: SLS Female with greater hip abduction concentric and hip extension eccentric strength had lower knee valgus angles SLL (FL, SML, SLL) Female with greater hip abduction concentric strength (very strong negative correlation) and hip abduction eccentric strength (strong negative correlation) had lower knee valgus angles.
Claiborne et al. 2006	F = 15 (23.5 ± 3.7y) M = 15 (26.4 ± 5.2y)	Isokinetic eccentric/concentric strength: Hip (abduction/adduction, flexion/extension, and internal/external rotation Knee valgus: FPKM (Frontal plane knee motion) in 3-D during SLS	BOTH: SLS Greater strength of concentric hip abduction among participant with lower knee valgus angles. (Weak to moderate negative correlation)
Suzuki 2015	F = 23 (19.96 ± 0.77y) M = 20 (20.20 ± 1.54y) intercollegiate basketball players	Hand-held dynamometer – hip extensor, abductor and external rotator Knee valgus 3-D analysis system during SLL (SML)	FEMALE: SLL Greater knee valgus angle at IC among participant with lower strength of hip extension, hip abduction and external rotation.
Stickler et al. 2015	F = 40 (22.88 ± 0.32y)	Handheld dynamometer isometric: hip abduction, extension, external rotation Knee valgus: FPPA (2-D) during SLS	FEMALE: SLS Female with greater hip abduction, hip extension and external rotation strength had lower knee peak valgus angles (FPPA)
Wilson et al. 2006	F = 22 (19.4 ± 0.7y) M = 24 (19.9 ± 2.3y) Division 1A or 1AA basketball, soccer, or volleyball players.	Peak isometric torque: Hip abduction and external rotation Knee valgus: FPPA (2-D) during SLS	BOTH: SLS Greater hip external rotation strength among participant with greater knee valgus angles (positive correlation)
Jacobs & Matacola 2005	F = 10 (22.1 ± 2.3y) M = 8 (24.1 ± 2.2y) Recreationally active adults.	Isokinetic peak eccentric torque: Hip abductor Knee valgus: 3-D analysis system during SLL	FEMALE: SLL Female with larger eccentric peak torque had lower peak knee valgus angles MALE: SLL No significant correlations between eccentric peak torque and knee valgus
ACTIVATION/COACTIVATION			
Mauntel et al. 2013	MKD group (20.2 ± 1.8y) F = 10 M = 10 Control group (20.2 ± 1.5y) F = 10 M = 10 physically active, defined as participation in at least 30 min. of physical activity, 3 times per week for at least 6 months	EMG Dynamic (descent phase of SLS) and MVIC Gluteus maximus and medius, hip adductors (hip coactivation ratios) Knee valgus: 3-D motion analysis during SLS	BOTH: SLS Hip coactivation ratios shows smaller gluteus medius to hip adductor (GMed:Hip Add) and gluteus maximus to hip adductor (GMax:Hip Add) coactivation ratios in valgus group than in the control group
Neamatallah 2020	F = 17 (25.7 ± 4.5y) M = 17 (26.9 ± 3.8y) physically active (participating in least 3 h of exercise per week)	EMG Dynamic and MVIC Gluteus maximus and medius Knee valgus: 3D motion analysis during SLS/SLL (Forward Landing, Single Medial Landing, Single Lateral Landing)	FEMALE: SLL (FL) Higher G Max EMG activity was associated with higher knee valgus angles among female (positive correlation). MALE: SLS: Higher G Med. EMG activity was associated with higher knee valgus angles among male (positive correlation).
ANKLE AND FOOT			
Study	Participants	Outcome parameters	Results
Wyndow et al. 2016	Both = 30 (22 ± 3y)	Ankle dorsiflexion Range: knee-to-wall lunge test Foot mobility was quantified as the difference in dorsal midfoot height or midfoot width, between non-weight bearing and bilateral weight bearing positions Knee valgus: 2-D FPPA during SLS	BOTH: SLS higher midfoot width mobility, or lower ankle joint dorsiflexion range and midfoot height mobility, were associated with a greater knee valgus angles (FPPA)
Mauntel et al. 2013	Valgus group (20.2 ± 1.8y) F = 10 M = 10 Control group (20.2 ± 1.5y) F = 10 M = 10 physically active, defined as participation in at least 30 min. of physical activity, 3 times per week for at least 6 months	Ankle dorsiflexion range (in extended position) Supine Leg straight – goniometer measurement (in flexed) Supine Knee flexed – goniometer measurement Knee valgus: 3-D motion analysis during SLS	BOTH: SLS Valgus group displayed significantly less passive ankle dorsiflexion with the knee extended and flexed
Kagaya et al. 2015	F = 130 (16.9 ± 0.6y) high-school basketball players, basketball experience, 6.7 ± 2.0 y.	Rear-foot eversion alignment – dynamic heel-floor test (HFT) Knee valgus: 2D video images (Knee-in distance) during SLS and SLL	FEMALE: SLS and SLL The KID were greater in the HFT-positive (≥5˚ angle) than in the HFT-negative group (<5˚ angle)

Abbreviations: ACL—Anterior Cruciate Ligament, PFP—patellofemoral pain, SLS—Single Leg Squat, SLL—Single Leg Landings, BOTH—both sex (female and male), FPKM—Frontal Plane Knee Motion, FPPA—Frontal Plane Projection Angle, MKD—Medial Knee Displacement, EMG—Electromyography, MVIC—Maximum Voluntary Isometric Contraction, HFT—Heel-fFlot Test, KID—Knee-In Distance.
